# Efficient resolution of racemic crown-shaped cyclotriveratrylene derivatives and isolation and characterization of the intermediate saddle isomer

**DOI:** 10.3762/bjoc.15.133

**Published:** 2019-06-18

**Authors:** Sven Götz, Andreas Schneider, Arne Lützen

**Affiliations:** 1 Kekulé-Institute of Organic Chemistry and Biochemistry, University of Bonn, Gerhard-Domagk-Str. 1, D-53121 Bonn, Germany

**Keywords:** chiral resolution, cyclotriveratrylenes, HPLC, macrocycles, racemization, saddle isomer

## Abstract

The preparative resolution of a trifunctionalized *C*_3_-symmetrical chiral cyclotriveratrylene derivative was achieved via high-performance liquid chromatography (HPLC) on a chiral stationary phase. This approach is a promising alternative to the previously reported resolution through formation of diastereomeric esters because it involves fewer synthetic steps and is less prone to thermal (re)racemization. During these studies an intermediate saddle conformer could also be isolated and characterized by ^1^H and ^13^C NMR spectroscopy. The HPLC separation method was further developed in order to allow investigations on the racemization behavior of the cyclotriveratrylene derivative.

## Introduction

Cyclotriveratrylenes (CTVs) [1–8] are cyclic bowl-shaped molecules and belong to the most studied concave host molecules in supramolecular chemistry besides, e.g., calixarenes and resorcinarenes [9], cyclodextrins [10–11] and cucurbiturils [12–13]. CTVs are cyclic trimers, originally formed from veratrole upon condensation with formaldehyde, which adopt a bowl-shaped form as the most stable conformation.

**Scheme 1:**
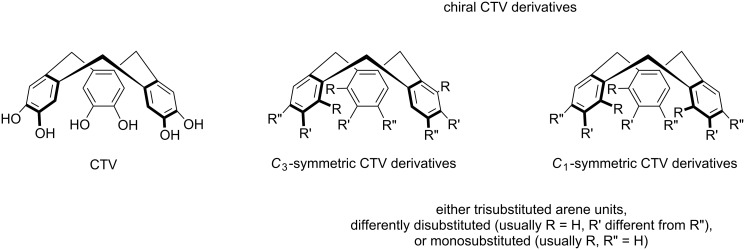
CTV and chiral CTV derivatives.

Chiral derivatives result when the aromatic residues carry either one, three or two different additional substituents which reduces the symmetry from *C*_3_*_v_* to either *C*_3_ or *C*_1_ (Scheme 1) [14–16].

Ever since their first synthesis [17–18] these chiral CTV derivatives have gained much attention. This is especially true for their covalently assembled dimers, the so called cryptophanes pioneered by A. Collet as they bear a chiral cavity that can be used for molecular recognition [19]. Probably the most famous example for this behavior is the chiral discrimination and determination of the absolute configuration of the two enantiomers of CHFClBr using an enantioenriched cryptophane-C [20–21]. Other examples are Xe inclusion complexes that are of special interest as they help to establish laser-polarized ^129^Xe NMR spectroscopy for the imaging in biological systems [22–25]. For the formation of Xe cryptophane complexes, cryptophane-1.1.1 has proven to be a very suitable host as it still shows the highest binding constant for Xe encapsulation in organic solvents known today [25].

However, unless fixed like in a cryptophane CTVs are usually flexible enough to undergo ring inversion which is synonymous to a racemization in case of chiral CTVs. This intramolecular process presumably proceeds via less stable saddle conformers. A first example of such a saddle isomer of an achiral hexamethoxy-substituted CTV could be isolated and described by Luz and co-workers in 2004 [26]. At that time, they achieved this by heating the crown form to high temperatures and subsequent rapid cooling of the mixture. Isolation of the product was then possible through repeated column chromatography. Similarly, the same group was also able to isolate the saddle stereoisomer of a chiral nonamethoxy-substituted CTV in which every arene unit carries three additional substituents through HPLC on a chiral stationary phase [27–28].

**Scheme 2:**
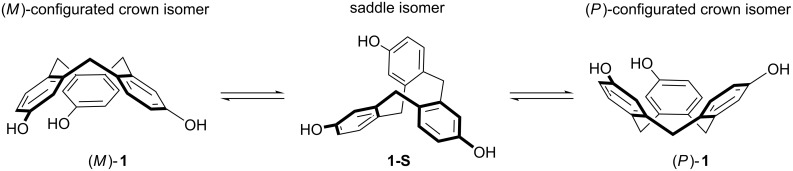
The two enantiomeric crown isomers of chiral CTV **1** and its saddle isomer **1-S**.

Due to our interest in dissymmetric [29–36] and concave molecular building blocks [37] and their implementation in supramolecular architectures like (allosteric) receptors [38–44] or metallosupramolecular helicates and cages [45–57] we were intrigued by the class of chiral CTVs. This is especially true for derivative **1** (Scheme 2) due to its interesting trifold substitution pattern with an almost orthogonal orientation of the functional groups which make it an ideal precursor for the synthesis of other elaborated derivatives [58–59] and the ease of a recently established large-scale synthesis reported by Rousseau and co-workers [60].

Hence, we decided to revisit this compound in order to improve the separation in terms of both better resolution of the enantiomeric bowl-shaped conformers and isolation of the intermediate saddle conformer.

## Results and Discussion

### Synthesis and chiral resolution of **1** and isolation of the saddle isomer

**Scheme 3:**
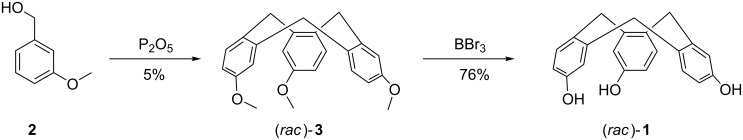
Synthesis of CTV **1**.

The synthesis of (*rac*)-**1** was accomplished according to the already mentioned protocol by Rousseau and co-workers (Scheme 3) [60] although it should be noted here that another route for the synthesis of (*rac*)-**3** was published by Dutasta and co-workers [61].

With the racemic materials in hands we then searched the literature for successful resolution protocols for chiral CTV derivatives. In fact, Collet and Gottarelli reported on the first resolution of (*rac*)-**1** already in 1984 [62]. At that time, they converted the racemic triol into diastereomeric triesters upon reaction with (*R*)-(+)-2-phenoxypropionic acid, separated those via column chromatography, and received the desired enantiomers upon reductive cleavage of the esters. Similar synthetic approaches through the formation of diastereomeric esters were also used for other chiral CTV derivatives [63–67]. However, performing the separation on the stage of a monomeric CTV always bears the risk of racemization because of the flexibility of these molecules and kinetic studies for several CTVs demonstrate that the energy barrier for the racemization in each case is around 110 kJ mol^−1^ [62,68–69]. Thus, the necessary synthetic steps to introduce the chiral auxiliary and cleave it after successful isolation of the pure diastereomers can only be performed under rather mild conditions in order to avoid racemization. This is why Collet and co-workers also developed alternative methods for the chiral resolution of other CTVs and cryptophanes via high-performance liquid chromatography (HPLC) on a CHIRALPAK OT(+) column as stationary phase already in the 1980s [70]. Later on other chiral CTVs could also be successfully separated on a Regis (*S*,*S*)-Whelk-O1 and a CHIRALCEL OD-H stationary phase [27–28,71].

**Figure 1:**
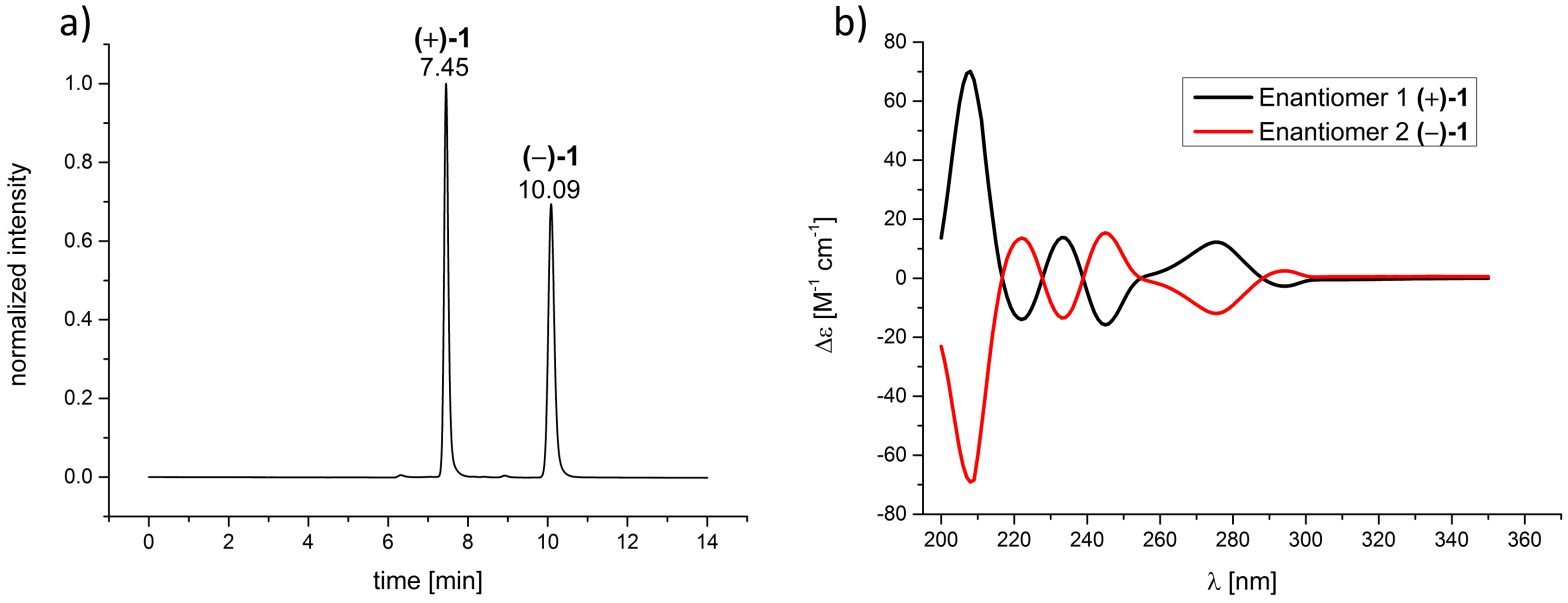
a) Chromatogram of an analytical separation of (*rac*)-**1** on a CHIRALPAK IB column as the stationary phase and MeOH as the mobile phase, 298 K, flow rate: 0.5 mL min^−1^; b) ECD-spectra of (+)-(*M*)-**1** and (−)-(*P*)-**1** measured in CH_3_CN; black: first eluted enantiomer (+)-(*M*)-**1** (*c* = 1.06 × 10^−4^ M), red: second eluted enantiomer (−)-(*P*)-**1** (*c* = 1.03 × 10^−4^ M) (assignment of the absolute configuration of the enantiomer according to the assignment of Collet and Gottarelli [62]).

Therefore, we also decided to employ an HPLC approach and for the first time we could achieve an effective resolution of CTV **1** using a CHIRALPAK IB column as the stationary phase and pure MeOH as the mobile phase (Figure 1). This simple procedure was successfully used for the semi-preparative separation of 156 mg of (*rac*)-**1**. It was possible to separate more than 25 mg substance per injection giving both enantiomers of **1** in an enantiomeric excess of >99%. Electronic circular dichroism spectra (ECD) were also recorded and are shown in Figure 1.

**Figure 2:**
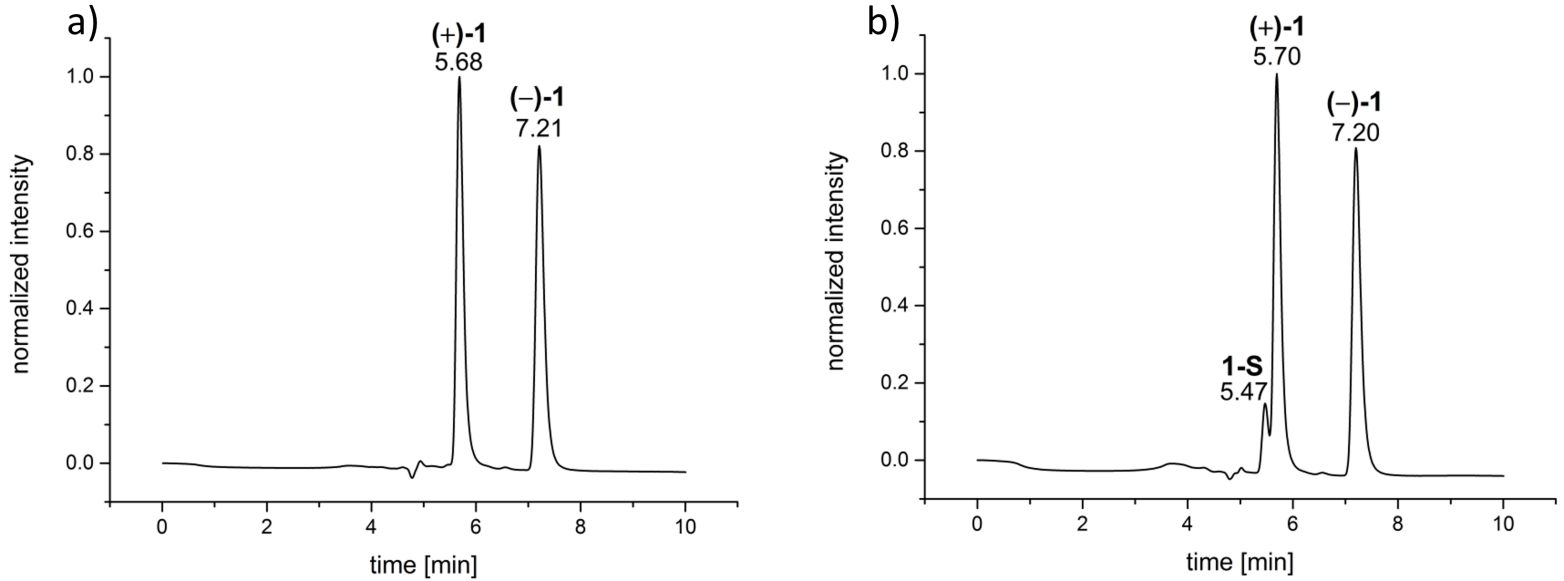
a) Chromatogram of the analytical separation of (*rac*)-**1** (CHIRALPAK IB, 100% MeOH, 293 K, flow rate: 0.7 mL min^−1^) before heating to 78 °C in EtOH; b) chromatogram of the analytical separation via HPLC of (*rac*)-**1** (CHIRALPAK IB, 100% MeOH, 293 K, 0.7 mL min^−1^) after heating to 78 °C in EtOH for 2 h. The new peak corresponds to the saddle conformer and is marked with **1-S**.

When we then started to examine the racemization behavior of **1** by heating solutions of (+)-(*M*)-**1** and (−)-(*P*)-**1** (assignment of the absolute configuration of the enantiomer according to the assignment of Collet and Gottarelli [62]) in EtOH to 78 °C and determining the ee values after different time intervals to plot the time course of the racemization process we made an interesting observation: the chromatogram changed upon heating as a shoulder appeared at the peak of the first enantiomer (Figure 2).

**Figure 3:**
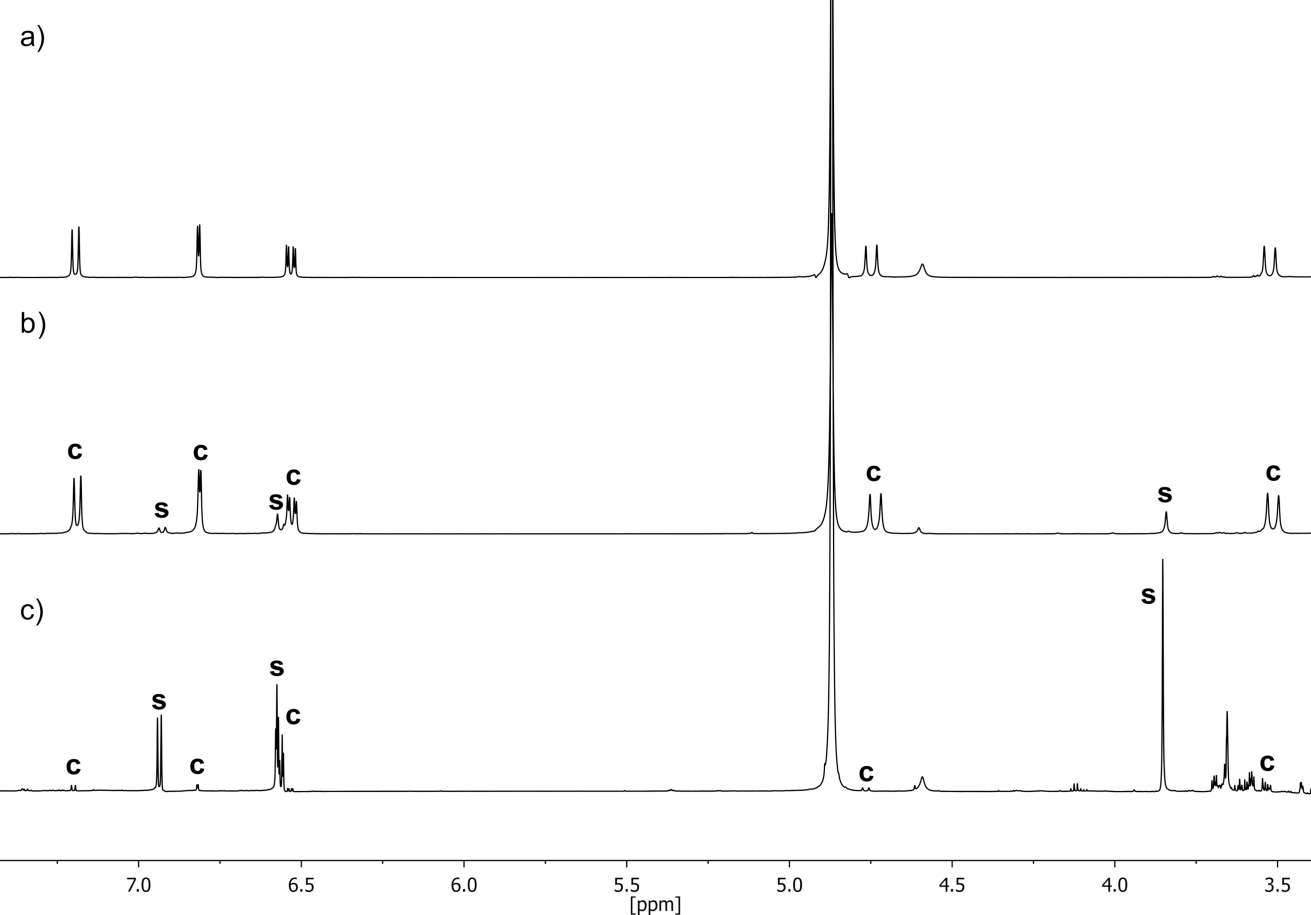
a) ^1^H NMR spectrum of the neat crown isomers of (*rac*)-**1** in CD_3_OD (400 MHz, 298 K); b) ^1^H NMR spectrum in CD_3_OD (400 MHz, 298 K) of a sample of (*rac*)-**1** after heating it to 200 °C in DMSO and quenching the solution by pouring it into an ice water mixture; c) ^1^H NMR spectrum of **1-S** in CD_3_OD (700 MHz, 298 K) after isolation through HPLC. The signals of the crown and the saddle isomers of **1** are marked with **C** and **S**, respectively.

As a possible explanation for this new species we considered the formation of the saddle isomer **1-S**. As stated above, Luz and colleagues were the first who could successfully separate such a saddle isomer of another chiral nonamethoxy-CTV [27–28]. Hence, we were wondering, if we could achieve the same for CTV **1**, too. To ensure that the new signal really belongs to the saddle isomer, its isolation and characterization was necessary. First, we tried to generate **1-S** by heating a DMSO solution of (*rac*)-**1** to 200 °C for 5 minutes and quenching the mixture by pouring it into ice water according to the procedure established by Luz for the methoxy-substituted CTV derivatives [26–28]. Unfortunately, probably because of its hydroxy groups, **1** proved to be moderately soluble in water, which caused a massive decrease in the amount of material that we were able to isolate in the end. Also, a separation through normal column chromatography on silica, as performed by Luz with an achiral hexamethoxy-substituted CTV derivative [26], was not successful. Therefore, we again turned to HPLC for the isolation of the new species via semi-preparative HPLC on a chiral stationary phase. This, however, asked for changing the conditions used for the chromatography compared to the approach described above which proved to be very effective to separate the two enantiomers. The ideal method should have a short retention time because less than 5% of the new species were formed upon heating. Such conditions were finally found using a semi-preparative (*S*,*S*)-Whelk O1 column as the stationary phase and a mixture of *n-*hexane/EtOH 70:30 as the eluent. Although there was no baseline separation of the two enantiomers of **1**, a retention time of 6.20 minutes of the anticipated saddle isomer **1-S** was the shortest we found (see Supporting Information File 1). Nevertheless, 30 injections were necessary to collect enough material to record sufficient ^1^H and ^13^C NMR spectra. The spectra were very similar to those published by Luz [26], thus verifying our assumption. Please note, that the saddle stereoisomer of a CTV undergoes very fast intramolecular pseudorotational motions to change between its six conformers. The equilibrium between these species is, in fact, so fast that the individual conformers cannot be isolated, even by cooling down to 100 K in a freon solvent as tried by Luz and co-workers for a nonamethoxy-CTV [27–28]. On the NMR time scale this results in an average *C*_3_*_h_*-symmetry of the compound which corresponds to a “*flat*” molecule where both protons of the methylene bridges are magnetically equivalent resulting in only one singlet in the ^1^H NMR (Figure 3) [27–28,72]. It is also important to mention that **1-S** is obviously not as stable in solution as the saddle isomer of the nonamethoxy-CTV, as we observed a significant amount of newly formed crown isomer immediately after HPLC separation at room temperature (21%). However, the amount of the crown isomer could by significantly decreased to 6% by cooling the solution of **1-S** at a temperature of −10 °C immediately after collecting it after HPLC separation. The fact, that the isolated saddle isomer forms both enantiomers, even at room temperature, underlines the proposed mechanism of racemization [1].

### Experiments on the racemization behavior of **1**

**Figure 4:**
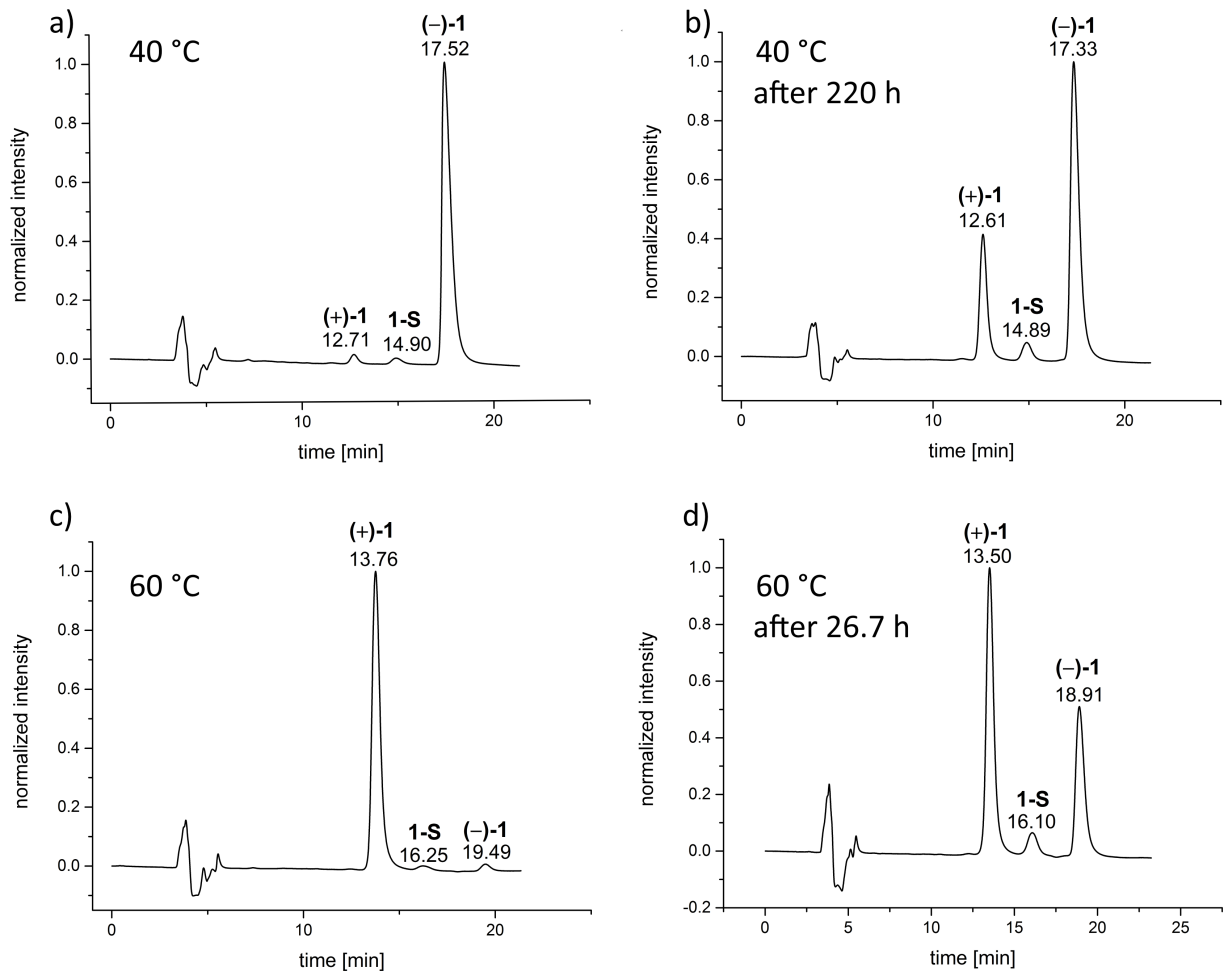
Chromatograms of the analytical separations (CHIRALPAK IB, acetonitrile/water 40:60, 293 K, flow rate: 0.85 mL min^−1^) obtained during the racemization experiments. a) and b) are from the series of experiments with samples heated to 40 °C in EtOH, starting with (−)-(*P*)-**1**, c) and d) are from the series of experiments with samples heated to 60 °C in EtOH, starting with (+)-(*M*)-**1**.

After successful isolation and characterization of the saddle isomer we returned to our initial goal to gain insight into the racemization behavior of **1**. With the knowledge, that the saddle isomer **1-S** is already existent at relatively low temperatures and that the racemization proceeds via the saddle form, we tried to perform additional experiments that allow us to determine the relative change of the ratios of the two enantiomers and the saddle isomer. To do so, we established yet another separation protocol, that allowed baseline separation of all three species, and hence, the direct determination of their ratio from the data that analytical HPLC provides. We found that a CHIRALPAK IB column as the stationary phase and a mixture of acetonitrile/water 40:60 as the eluent fulfilled these requirements even though the retention times became relatively long (Figure 4).

**Figure 5:**
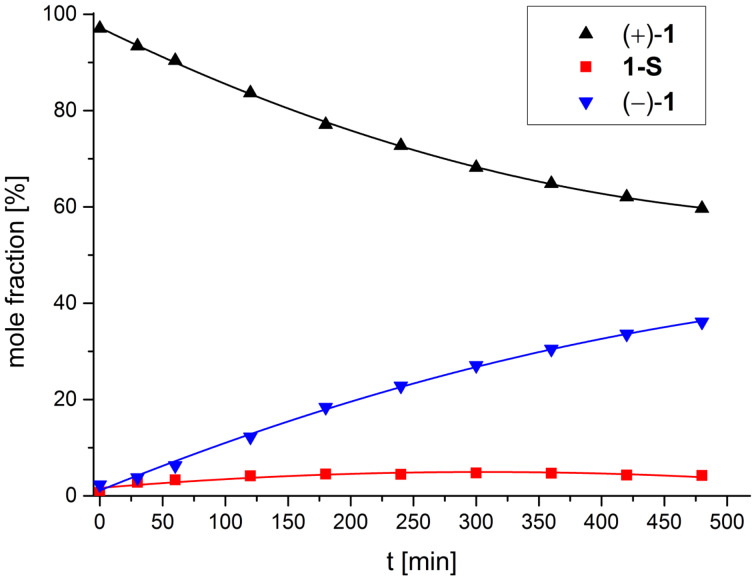
The mole fractions obtained in the racemization experiment plotted against the time, with black triangles for (+)-(*M*)-**1**, blue triangles for (−)-(*P*)-**1** and red squares for **1-S**. The experiment at a temperature of 70 °C starting with (+)-(*M*)-**1** is shown.

For the racemization experiment solutions of (+)-(*M*)-**1** or (−)-(*P*)-**1** in EtOH were heated to 40, 50, 60 or 70 °C, respectively and after certain time intervals, 10 µL samples of the solution were analyzed by HPLC. The ratio of the three species was determined by integration and the data were plotted against the time. A representative graph for a racemization experiment at 70 °C is shown in Figure 5.

In all experiments the amount of **1-S** is nearly constant over the course of the racemization, e.g., at 40 °C there are about 3% and at 70 °C about 4.5% of **1-S** present in solution (see Supporting Information File 1).

In the past, Collet determined rate constants for the interconversion of **1** under the assumption of first order kinetics. Although the presence of the in-between lying saddle isomer indicates a more complex kinetic, we decided, for the sake of comparability, to determine rate constants and energy barrier Δ*G*^≠^ for the racemization also with the assumption of first order kinetics (see Supporting Information File 1 for details). Analysis of the data gave a racemization barrier of 114.3 kJ mol^−1^ which is in good agreement with the value of 114.0 kJ mol^−1^ reported by Collet for his experiments in dioxane [62]. Perhaps equally interesting for anyone planning to work with this compound this translates into the following half-life values for **1** (defined as the time when the ee value is 50): 4.8 days at 40 °C, 2 days at 50 °C, 8.3 hours at 60 °C and 3.2 hours at 70 °C.

## Conclusion

In summary, we were able to establish an efficient HPLC protocol using a chiral stationary phase for the chiral resolution of CTV **1** which is a key compound for the synthesis of cryptophanes but also a very promising building block for the synthesis of other sophisticated (supra)molecular architectures. Using a CHIRALPAK IB as the stationary phase and MeOH as the mobile phase, it is possible to resolve more than 100 mg of racemic material with only a few injections in about 1.5 h. That makes this method a competitive alternative for the separation of lab-scale amounts of the enantiomers of **1** compared to the separation through formation of diastereomeric esters.

While we tried to investigate the racemization behavior, we observed the formation of the less stable saddle isomer **1-S**, which we could then even isolate and characterize by ^1^H NMR and ^13^C NMR spectroscopy. Furthermore, we developed another new HPLC protocol that allowed baseline separation of both enantiomers and the saddle isomer. This enabled us to determine the relative ratios of these three species directly, and hence, to follow the racemization process of **1**. Following this approach, we determined an energy barrier of the racemization of **1** of 114.3 kJ mol^−1^ which is in good agreement with the literature data. Hence, the racemization of **1** has half-life times ranging from a couple of days at 40 °C to only a few hours at 70 °C in ethanol which sets the limits for further derivatization.

## Supporting Information

The supporting information contains the synthetic protocols for the preparation of CTV **1** and the characterization of the crown isomers and the saddle isomer including the corresponding spectra, if not already shown in the main article. It also contains details of the chromatographic protocols and the analysis of the racemization process.

File 1 Additional experimental details and spectra.
