# Supplementary material for: Efficient resolution of racemic crown-shaped cyclotriveratrylene derivatives and isolation and characterization of the intermediate saddle isomer
Source: Beilstein J Org Chem. 2019 Jun 18;15:1339–46. doi: 10.3762/bjoc.15.133 (PMC6604736; doi:10.3762/bjoc.15.133)
Supplement: File 1 — Additional experimental details and spectra. [file Beilstein_J_Org_Chem-15-1339-s001.pdf]

BEILSTEIN JOURNAL OF ORGANIC CHEMISTRY

Supporting Information

for

Efficient resolution of racemic crown-shaped cyclotriveratrylene derivatives and isolation and characterization of the intermediate saddle isomer

Sven Götz, Andreas Schneider and Arne Lützen

Beilstein J. Org. Chem. **2019,** *15,* 1339–1346. doi:10.3762/bjoc.15.133

Additional experimental details and spectra

License and Terms: This is a supporting information file under the terms of the Creative Commons Attribution License (http://creativecommons.org/licenses/by/4.0). Please note that the reuse, redistribution and reproduction in particular requires that the authors and source are credited.
The license is subject to the *Beilstein Journal of Organic Chemistry* terms and conditions: (https://www.beilstein-journals.org/bjoc)

Table of Contents

1. Synthetic procedures S2

2. HPLC separations S5

3. Kinetics of racemization S9

4. NMR spectra of (±)-**1** and **1-S** S20

1. Synthetic procedures

2,7,12-Trimethoxy-10,15-dihydro-5*H*-tribenzo[*a*,*d*,*g*]cyclononene (3)

A suspension of phosphorus pentoxide (9.77 g, 68.81 mmol) in 80 mL of dichloromethane was heated to 40 °C. Then, 3-methoxybenzyl alcohol (**2**, 18.86 g, 136.50 mmol) was added and the mixture was refluxed for 1 hour. The mixture was filtrated and the residue extracted three times with dichloromethane. The extract was concentrated under reduced pressure and combined with the filtrate. The solution was filtrated over a 50 g pad of silica and the filtrate was concentrated under reduced pressure. After the addition of 5 mL of diethyl ether, the solution was kept overnight at 0 °C. The white precipitate was filtrated, washed with 5 × 1 mL of diethyl ether and dried in high vacuum. The product (0.760 mg, 2.11 mmol, 5%) was obtained as a white solid. The analytical data are in accordance with those reported in the literature.[1]

10,15-Dihydro-5*H*-tribenzo[*a*,*d*,*g*]cyclononene-2,7,12-triol ((*rac*)-1)

Trimethoxycyclotriveratrylene **3** (3.43 g, 10.77 mmol) was suspended in 16 mL of dry dichloromethane and cooled to 0 °C. Boron tribromide (17.29 g, 69 mmol as solution in dichloromethane) was slowly added and the mixture was stirred overnight at room temperature. The reaction was quenched by the addition of an ice water mixture and brought to pH 6 with a saturated aqueous sodium carbonate solution. The residue was washed with 5 × 10 mL of hot water. The residue was dissolved in 12 mL of acetonitrile and treated with supersonic for 40 minutes. The formed precipitate was filtrated, washed with 5 × 5 mL of acetonitrile and recrystallized from acetonitrile. The product (2.30 g, 7.24 mmol, 76%) was obtained as white solid. The analytical data are in accordance with those reported in the literature.[1]

---

1 Traoré, T.; Delacour, L.; Kotera, N.; Merer, G.; Buisson, D.-A.; Dupont, C.; Rousseau, B.*Org. Process Res. Dev.* **2011**, *15*, 435–437, DOI: 10.1021/op100260w.

10,15-Dihydro-5*H*-tribenzo[*a*,*d*,*g*]cyclononene-2,7,12-triol (saddle isomer, 1-S)

A solution of 71 mg (*rac*)-**1** in 250 µL of EtOH was heated to 78 °C for 1 hour. The saddle isomer is then separated via HPLC on an (*S*,*S*)-Whelk-O1 column as the stationary phase and *n*-hexane/EtOH 70:30 as the mobile phase with a flow rate of 5.0 mL/min. The retention time of **1-S** was 6.20 minutes.

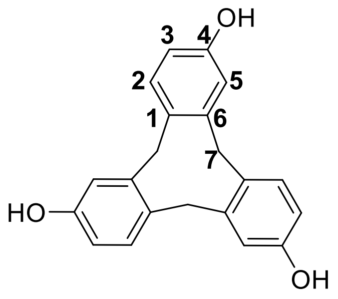

^{1}H-NMR (700,41 MHz, CD_3OD, 298 K, δ in ppm):

6.93 (d, 3 H, $^{3}J_{2,3}$= 8.1 Hz, H-2); 6,58 (d, 3 H, $^{3}J_{3,5}$= 2.5 Hz, H-5); 6,57 (dd, 3 H, $^{3}J_{5,3}$=2,5 Hz, $^{3}J_{2,3}$=8,1 Hz, H-3); 3,85 (s, 6 H, H-7).*[1]

^{13}C-NMR (176,14 MHz, CD_3OD, 298 K, δ im ppm):

156,7 (C-4); 141,9 (C-6); 132,4 (C-2); 131,3 (C-1), 117,9 (C-5); 114,2 (C-3); 39,1 (C-7).*[1]

*[1] The NMR spectra of **1-S** contained impurities, especially in the aliphatic region that derived from the tubes that were used to collect the product after the HPLC separation. These impurities were not analyzed in detail.

MS (EI) m/z (Intensity%): 318.1 (70), 211.0 (100).

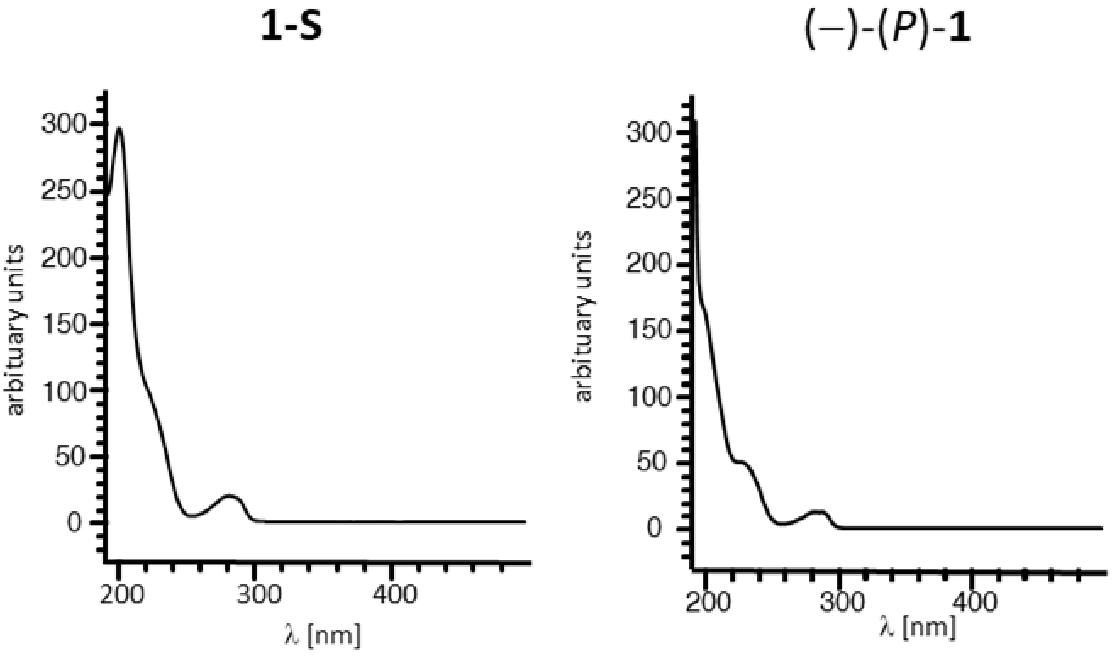

Figure S1: UV–vis spectra of solutions of **1-S** (left) and (−)-(*P*)-**1** (right) in a mixture of water/acetonitrile 60:40.

2. HPLC separations

Chiral resolutions of **1** were performed on an analytical ultra-high pressure gradient liquid chromatography system (UHPLC) from the *PLATINblue* series from *Knauer* and a semi-preparative system from the *Smartline* series from *Knauer*.

The isolation of **1-S** and the racemization experiments were performed with a *Prominence* system from *Shimadzu*. The columns that were used were an analytical CHIRALPAK IB (5 µm, 4.6 × 250 mm, with and without pre-column 4 × 10 mm), a semi-preparative CHIRALPAK IB (5 µm, 20 × 250 mm with pre-column 30 × 10 mm), an analytical (S,S)-Whelk O1 from *Regis Technologies* (10 µm, 4 × 300 mm) and a semi-preparative (S,S)-Whelk O1 from *Regis Technologies* (10 µm, 10 × 250 mm with pre-column 8 × 30 mm).

Chiral resolution of (*rac*)-1

- 156 mg of (*rac*)-**1** were dissolved in 1000 µL of MeOH and 400 µL of acetone.
- Separation on a semi-preparative CHIRALPAL IB column, 100% MeOH as the mobile phase, flow rate: 10 mL/min, UV detection at 230 nm.
- First enantiomer was eluted after 6.88 minutes,

  ee > 99%, $[\alpha]_D^{20}$ = +272 mL dm^{-1} g^{-1} (1.0 g/L, acetonitrile), (+)-(*M*)-**1**

  ECD: *λ* [nm] (*Δε* [mol L^{-1} cm^{-1}]): 208 (+70); 222 (–14); 233 (+14); 245 (–16); 275 (+12); 294 (–3).
- second enantiomer was eluted after 9.29 minutes,

  ee > 99%, $[\alpha]_D^{20}$ = –282° mL dm^{-1} g^{-1} (1.0 g/L, acetonitrile), (–)-(*P*)-**1**

  ECD: *λ* [nm] (*Δε* [mol L^{-1} cm^{-1}]): 208 (–69); 222 (+14); 233 (–13); 245 (+15); 275 (–12); 294 (+3).

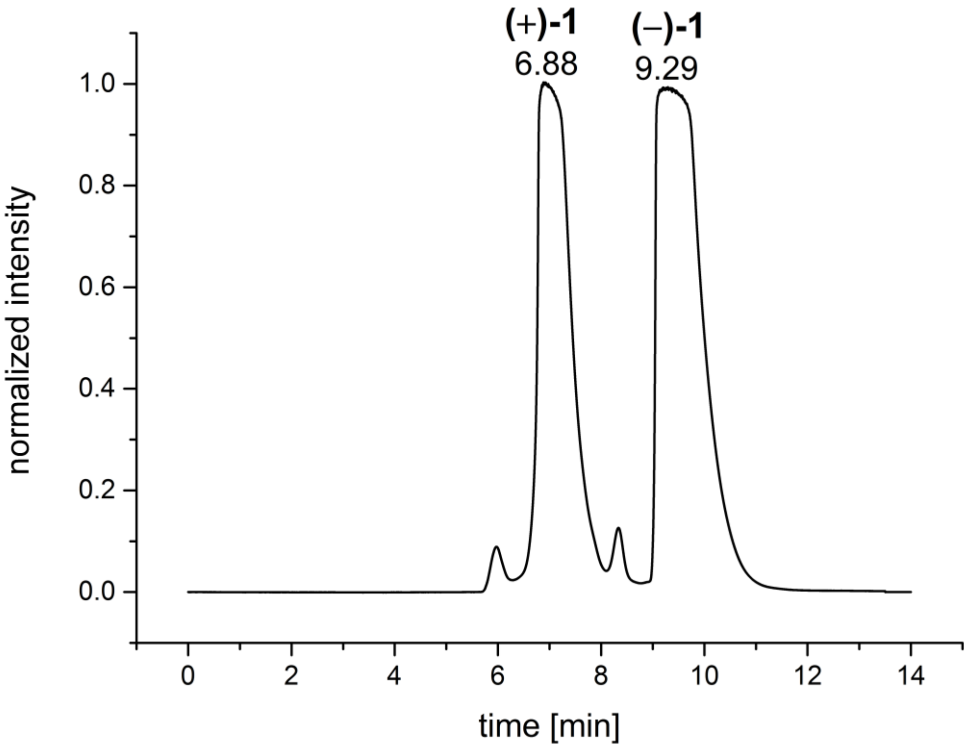

Figure S2: Chromatogram of a preparative separation of (*rac*)-**1** on a CHIRALPAK IB column as the stationary phase and MeOH as the mobile phase.

a)

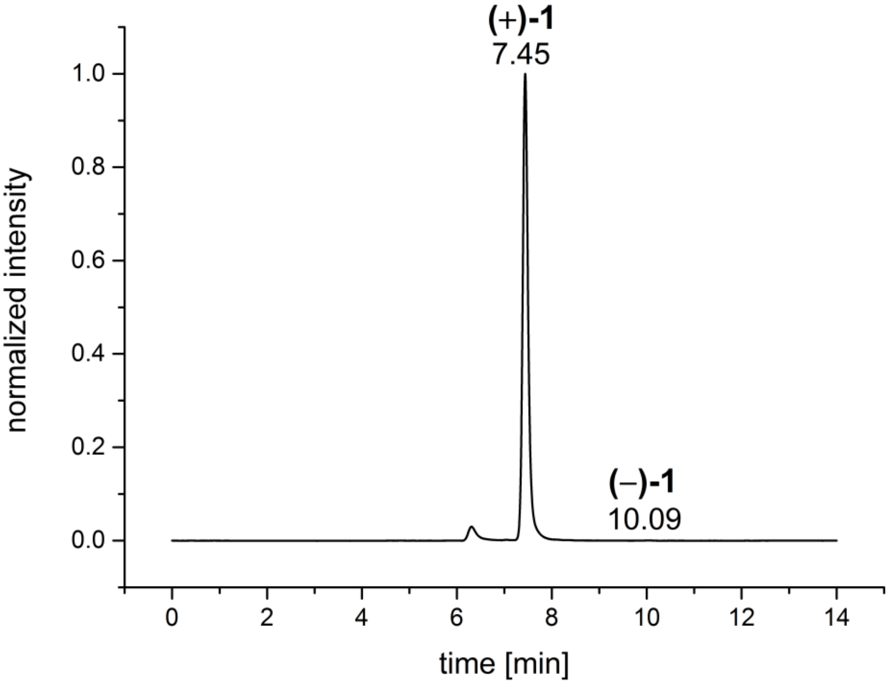

b)

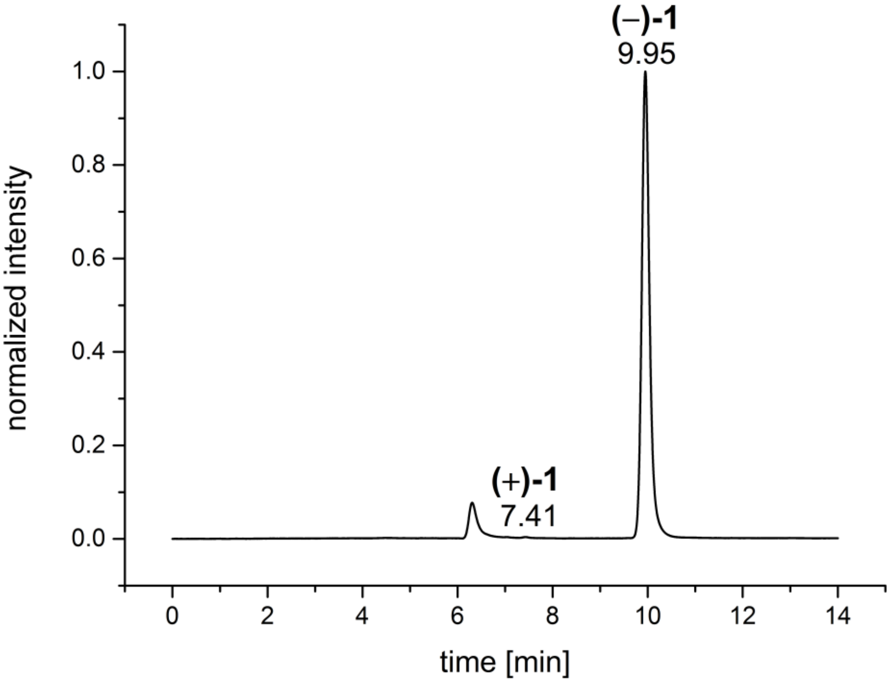

Figure S3: Chromatograms of analytical UHPLC runs of resolved enantiomers (CHIRALPAK IB column as the stationary phase and MeOH as the mobile phase): a) (+)-(*M*)-1, b) (−)-(*P*)-**1** (the compound eluting at 6.2 min is not one of the enantiomers).

Isolation of 1-S

- 71 mg were dissolved in 250 µL of EtOH and filtrated with a syringe filter.
- Separation on a semi-preparative (*S*,*S*)-Whelk-O1 column, *n*-hexane/EtOH 70:30 as the mobile phase, flow rate: 5.0 mL/min, UV detection at 254 nm.
- The retention time of **1-S** was 4.20 minutes.

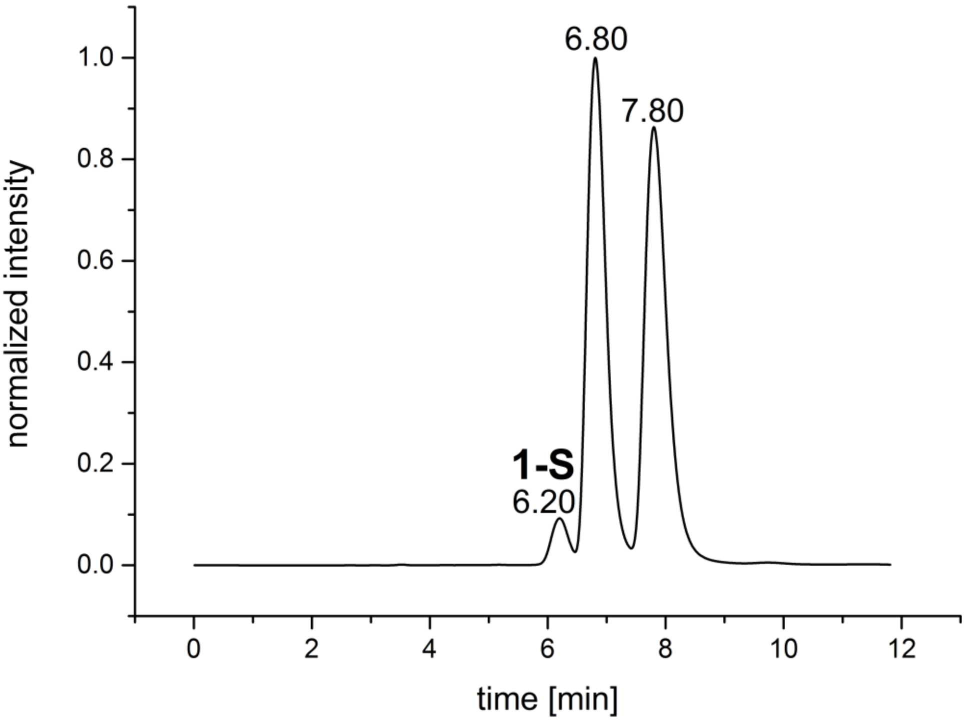

Figure S4: of a semi-preparative separation of (*rac*)-**1** on an (*S*,*S*)-Whelk O1 column as the stationary phase and *n*-hexane/EtOH 70:30 as the mobile phase.

3. Kinetics of racemization

Racemization experiments with (+)-1 and (−)-1

About 1 mg of (+)-**1** or (−)-**1** were dissolved in 1 mL of EtOH and the starting ee value was determined by HPLC. Then, the sample was placed in a preheated oil bath and heated to desired temperature. After the given time intervals, 10 µL of the solution were injected onto the CHIRALPAK IB column and the ee values were determined by integration.

Table S1: Data received from the racemization experiment starting with (+)-(*M*)-**1** in EtOH at 40 °C.

time [min]	time [h]	(+)-(*M*)-1 [%]	1-S [%]	(−)-(*P*)-1 [%]
0	0	97.37	0.81	1.82
82	1.37	96.88	0.85	2.27
1414	23.57	93.24	2.13	4.63
4179	69.65	84.53	2.94	15.33
5520	92.00	80.58	2.93	16.9
7132	118.87	77.57	2.85	19.58
8347	139.12	74.77	2.89	22.34
9872	164.53	71.68	3.01	25.31
15569	259.48	62.99	2.99	34.02
17349	289.15	61.18	3.01	35.81
21275	0.35	57.19	3.08	39.73

Table S2: Data received from the racemization experiment starting with (−)-(*P*)-**1** in EtOH at 40 °C.

time [min]	time [h]	(+)-(*M*)-1 [%]	1-S [%]	(−)-(*P*)-1 [%]
0	0	2.12	1	96.88
1476	24.60	3.71	2.56	93.73
8821	147.02	20.17	3.10	76.73
10061	167.68	22.04	3.09	74.87
11925	198.75	25.15	3.06	71.79
15869	264.48	31.52	3.05	65.43
20170	336.17	35.11	3.06	61.83
23080	384.67	37.15	2.95	59.90

Table S3: Data received from the racemization experiment starting with (+)-(*M*)-**1** in EtOH at 50 °C.

time [min]	(+)-(*M*)-1 [%]	1-S [%]	(−)-(*P*)-1 [%]
0	97.13	0.11	2.76
60	96.36	1.03	2.61
180	94.08	2.05	3.87
390	91.97	2.48	5.55
1500	80.26	2.89	16.85
1740	77.58	3.19	19.23
3435	75.35	3.44	21.2
4680	66.54	3.36	30.1

Table S4: Data received from the racemization experiment starting with (−)-(*P*)-**1** in EtOH at 50 °C.

time [h]	(+)-(*M*)-1 [%]	1-S [%]	(−)-(*P*)-1 [%]
0	1.69	0	98.31
60	2.28	0.73	96.98
180	2.83	1.91	95.26
390	4.79	2.46	92.75
1500	14.27	3.01	82.72
1740	16.22	3.57	80.21
3435	18.52	3.41	78.07
4680	27.4	3.53	69.07

Table S5: Data received from the racemization experiment starting with (+)-(*M*)-**1** in EtOH at 60 °C.

time [min]	(+)-(*M*)-1 [%]	1-S [%]	(−)-(*P*)-1 [%]
0	98.06	0.23	1.71
107	96.76	1.03	2.21
164	92.95	2.76	4.28
235	90.24	3.45	6.30
314	88.21	3.09	8.70
393	84.74	3.53	11.74
532	79.73	4.07	16.2
614	77.50	4.21	18.29
1058	60.47	3.49	36.04
1232	58.72	3.44	37.84
2540	50.57	3.81	45.62

Table S6: Data received from the racemization experiment starting with (−)-(P)-**1** in EtOH at 60 °C.

**time [min]**	**(+)-(*M*)-1 [%]**	**1-S [%]**	**(−)-(*P*)-1 [%]**
0	1.45	0.51	98.04
47	1.95	2.00	96.05
119	3.07	2.54	94.39
194	5.20	3.24	91.56
270	7.11	3.57	89.32
365	9.95	4.36	85.69
487	12,86	3.70	83.44
585	15.60	3.83	80.57
1604	32.39	3.75	63.86
1885	35.47	3.77	60.76
3141	43.20	3.66	53.14

Table S7: Data received from the racemization experiment starting with (+)-(*M*)-**1** in EtOH at 70 °C

**t [min]**	**(+)-(*M*)-1 [%]**	**1-S [%]**	**(−)-(*P*)-1 [%]**
0	97.06	0.66	2.28
30	93.41	2.82	3.77
60	90.39	3.32	6.28
120	83.66	4.12	12.22
180	77.11	4.51	18.38
240	72.72	4.47	22.81
300	68.18	4.75	27.07
360	64.85	4.69	30.47
420	62.07	4.32	33.61
480	59.7	4.22	36.09

Table S8: Data received from the racemization experiment starting with (−)-(*P*)-**1** in EtOH at 70 °C

**time [min]**	**(+)-(*M*)-1 [%]**	**1-S [%]**	**(−)-(*P*)-1 [%]**
0	1.89	1.06	97.05
60	6.7	4.26	89.03
120	11.78	4.73	83.49
180	17.05	4.83	78.12
240	21. 41	4.92	73.67
300	24.62	4.63	70.75
360	28.39	4.78	66.83
420	30.79	4.42	64.8
480	33.76	4.55	61.69

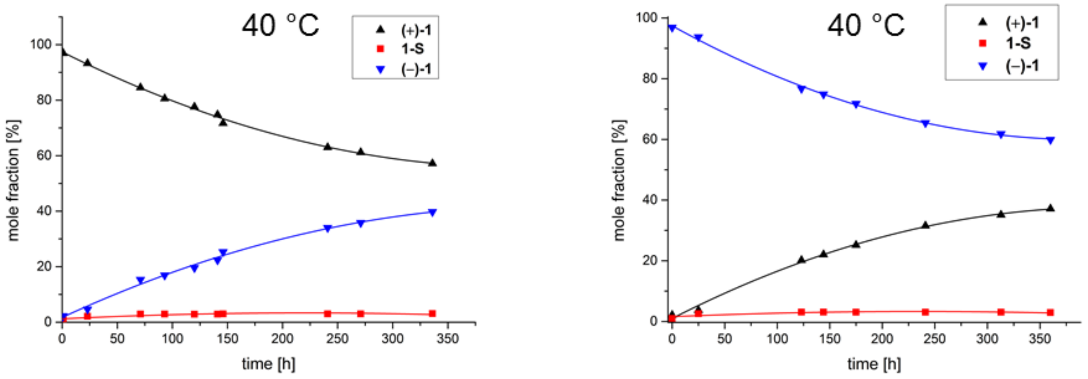

Figure S5: The mole fraction obtained in the racemization experiment plotted against the time, with black triangles for (+)-(*M*)-**1**, blue triangles for (−)-(*P*)-**1** and red squares for **1-S**. Both experiments were performed at 40 °C, left starting with (+)-(*M*)-**1** and right with (−)-(*P*)-**1**.

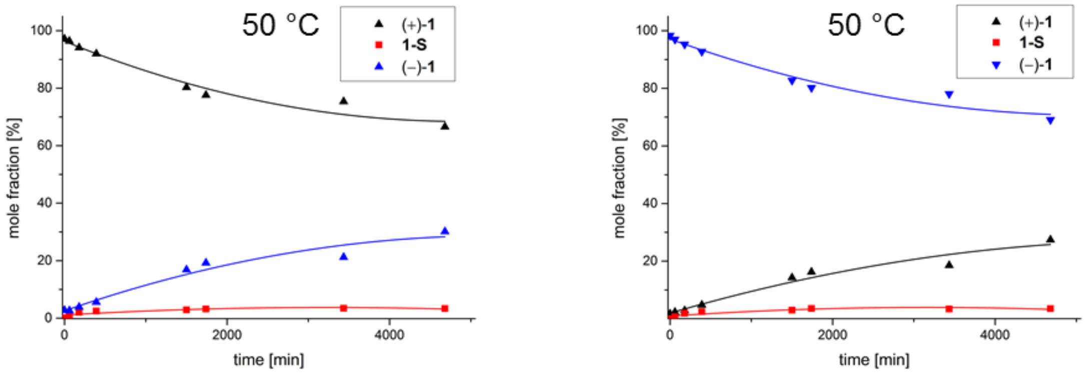

Figure S6: The mole fraction obtained in the racemization experiment plotted against the time, with black triangles for (+)-(*M*)-**1**, blue triangles for (−)-(*P*)-**1** and red squares for **1-S**. Both experiments were performed at 50 °C, left starting with (+)-(*M*)-**1** and right with (−)-(*P*)-**1**.

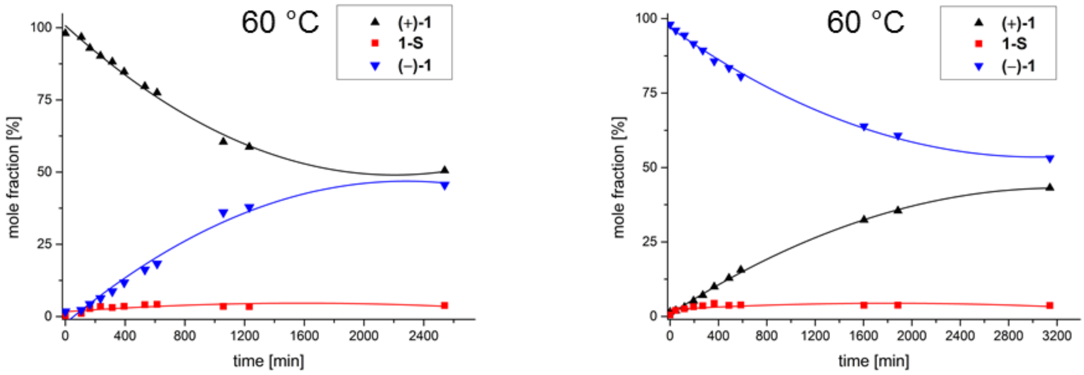

Figure S7: The mole fraction obtained in the racemization experiment plotted against the time, with black triangles for (+)-(*M*)-**1**, blue triangles for (−)-(*P*)-**1** and red squares for **1-S**. Both experiments were performed at 60 °C, left starting with (+)-(*M*)-**1** and right with (−)-(*P*)-**1**.

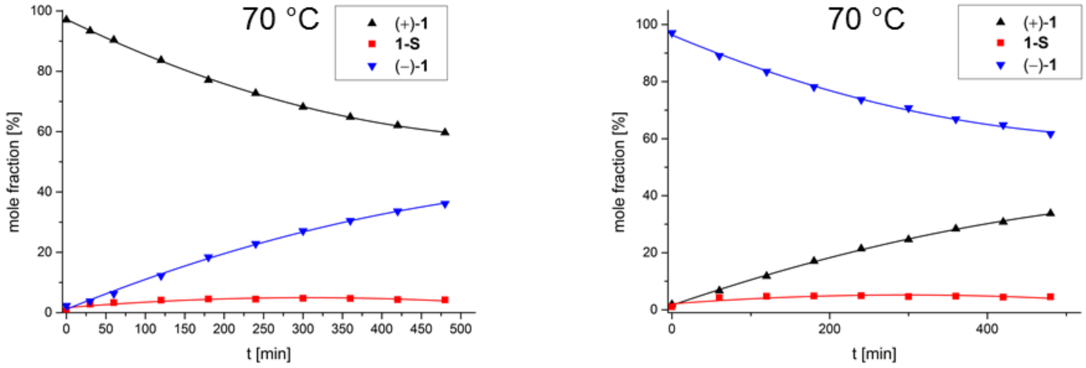

Figure S8: The mole fraction obtained in the racemization experiment plotted against the time, with black triangles for (+)-(*M*)-**1**, blue triangles for (−)-(*P*)-**1** and red squares for **1-S**. Both experiments were performed at 70 °C, left starting with (+)-(*M*)-**1** and right with (−)-(*P*)-**1**.

Determination of the rate constants and half-lifes

The values for k_{rac} were obtained by plotting the time against ln[((%(starting enantiomer) − 50%)/(%(starting enantiomer, t = 0) − 50%)]. The rate constants k_{rac} were calculated from the slopes according to the equation:

$$k_{rac} = -\frac{m}{2}$$

With: m: slope

The half-lifes $t_{1/2}$ were calculated according to the following equation

$$t_{1/2} = \frac{\ln\frac{4}{3}}{k_{rac}}$$

a) (+)-(*M*)-**1**, 40 °C

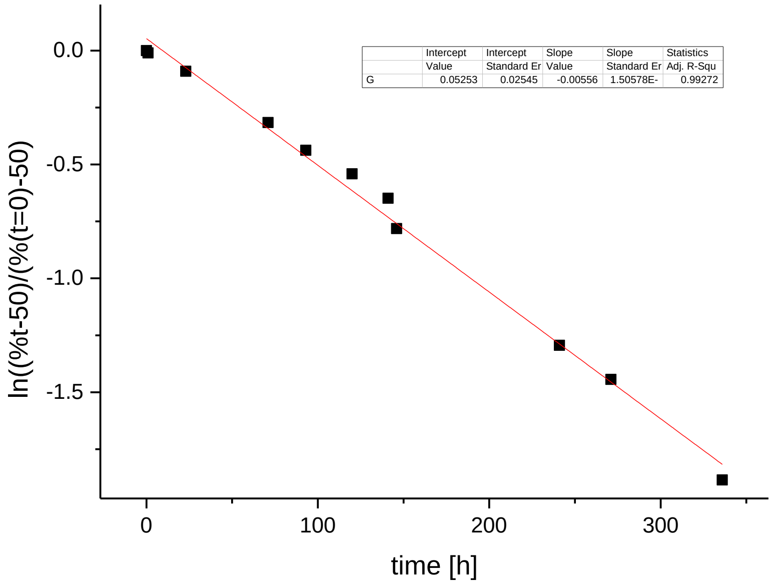

	Intercept	Intercept	Slope	Slope	Statistics
	Value	Standard Er	Value	Standard Er	Adj. R-Squ
G	0.05253	0.02545	-0.00556	1.50578E-	0.99272

$k_{rac} = 7.722 \times 10^{-7}\ s^{-1}$

$t_{1/2} = 6209.14$ min

b) (−)-(*P*)-**1**, 40 °C

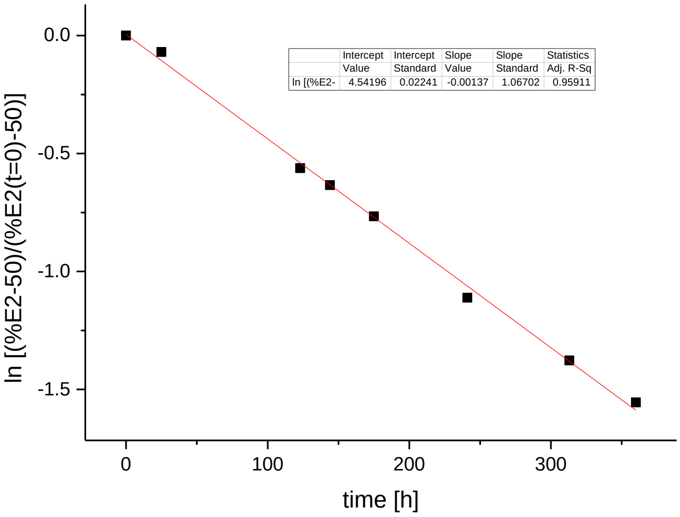

k_{rac} = 6.153 × 10^{-7} s^{-1}

$t_{1/2}$ = 7792.46 min

c) (+)-(*M*)-**1**, 50 °C

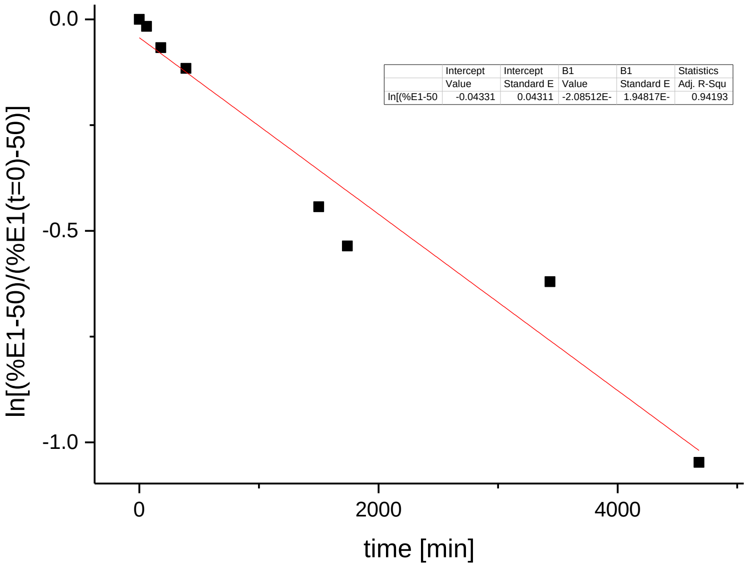

k_{rac} = 1.7376 × 10^{-6} s^{-1}

$t_{1/2}$ = 2759.38 min

d) (−)-(*P*)-**1**, 50 °C

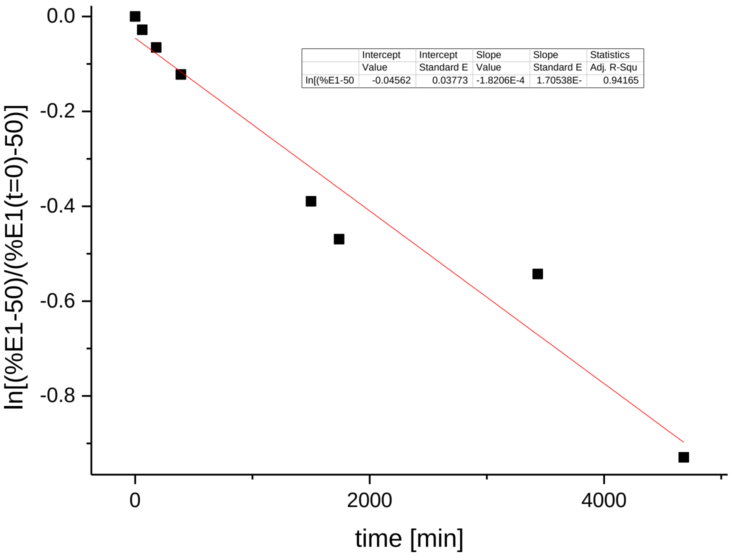

k_{rac} = 1.5171 × 10^{-6} s^{-1}

$t_{1/2}$ = 3160.44 min

e) (+)-(*M*)-**1**, 60 °C

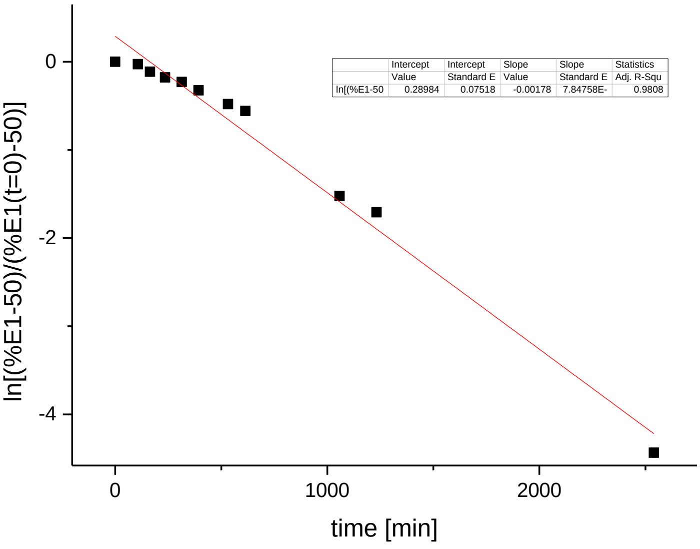

k_{rac} = 1.4833 × 10^{-5} s^{-1}

$t_{1/2}$ = 323.24 min

f) (−)-(*P*)-**1**, 60 °C

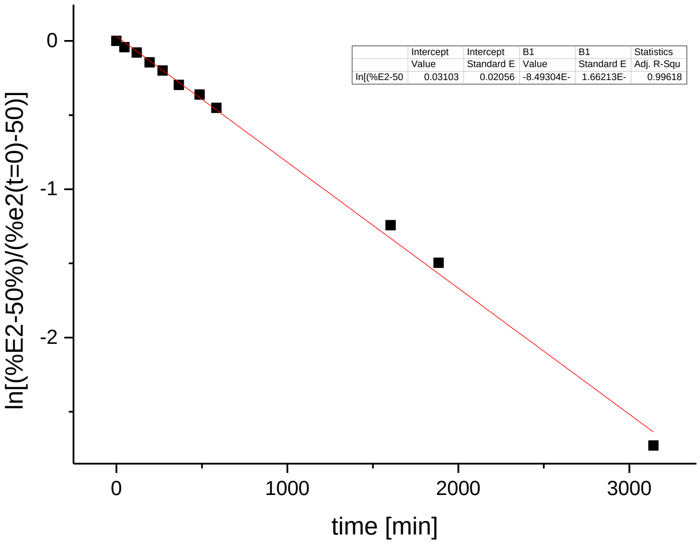

k_{rac} = 7,0775 × 10^{-6} s^{-1}

$t_{1/2}$ = 677.46 min

g) (+)-(*M*)-**1**, 70 °C

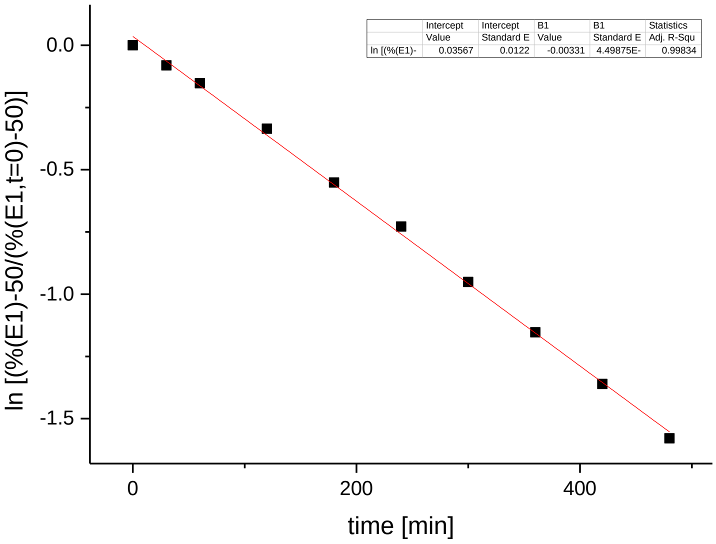

k_{rac} = 2.758 × 10^{-5} s^{-1}

$t_{1/2}$ = 173.85 min

h) (−)-(*P*)-**1**, 70 °C

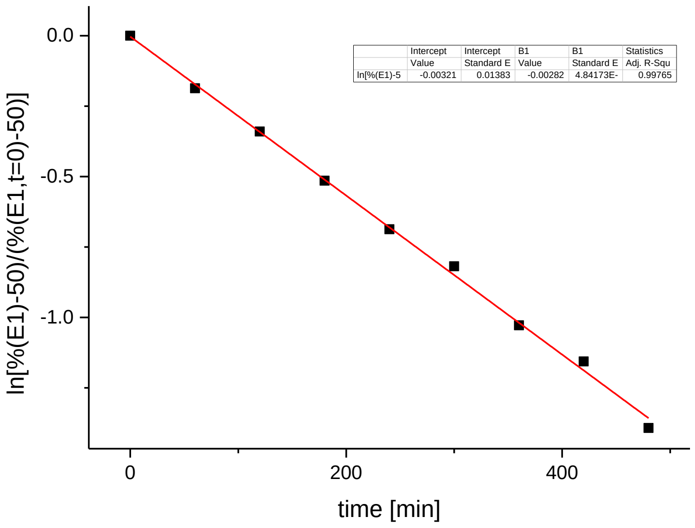

$$k_{\mathrm{rac}} = 2.35 \times 10^{-5}\ \mathrm{s}^{-1}$$

$$t_{1/2} = 204.03\ \mathrm{min}$$

Determination of the activation energy of racemization

The activation energy for the racemization $\Delta G^{\neq}$ was obtained by plotting ln(k_{rac}) against T^{-1}. For k_{rac} the average values for both enantiomers were used. $\Delta G^{\neq}$ was calculated according to the equation:

$$\Delta G^{\neq} = -m \cdot R$$

With: m: slope

R: universal gas constant

Table-S 9: Values for T−1 and ln(k) for the Arrhenius plot.

T [K]	1/T [K^{-1}]	ln($k_{average}$)
313.15	3.19E-03	-14.2179
323.15	3.09E-03	-13.3286
333.15	3.00E-03	-11.4217
343.15	2.91E-03	-10.5867

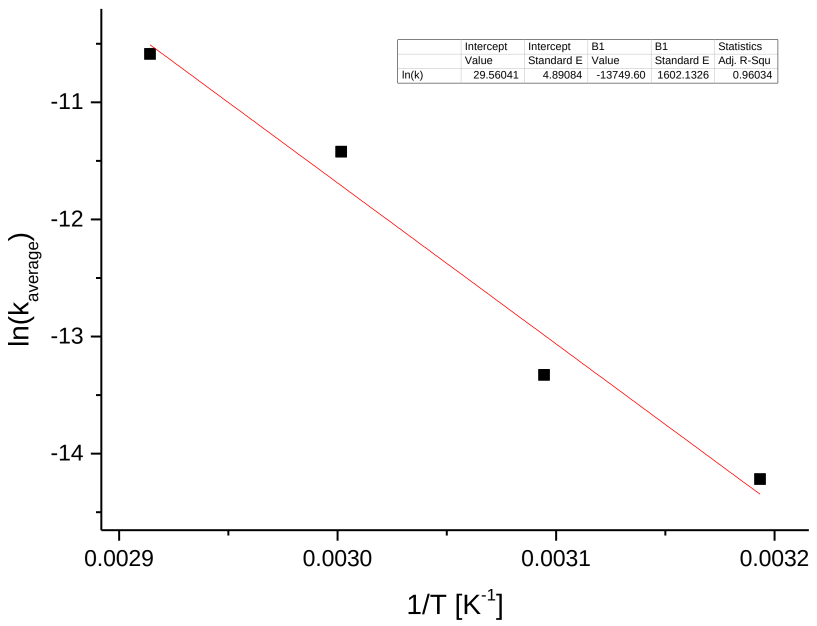

$\Delta G^{\neq}$ = 114.31 kJ mol^{-1}

4. NMR spectra of (±)-1 and 1-S

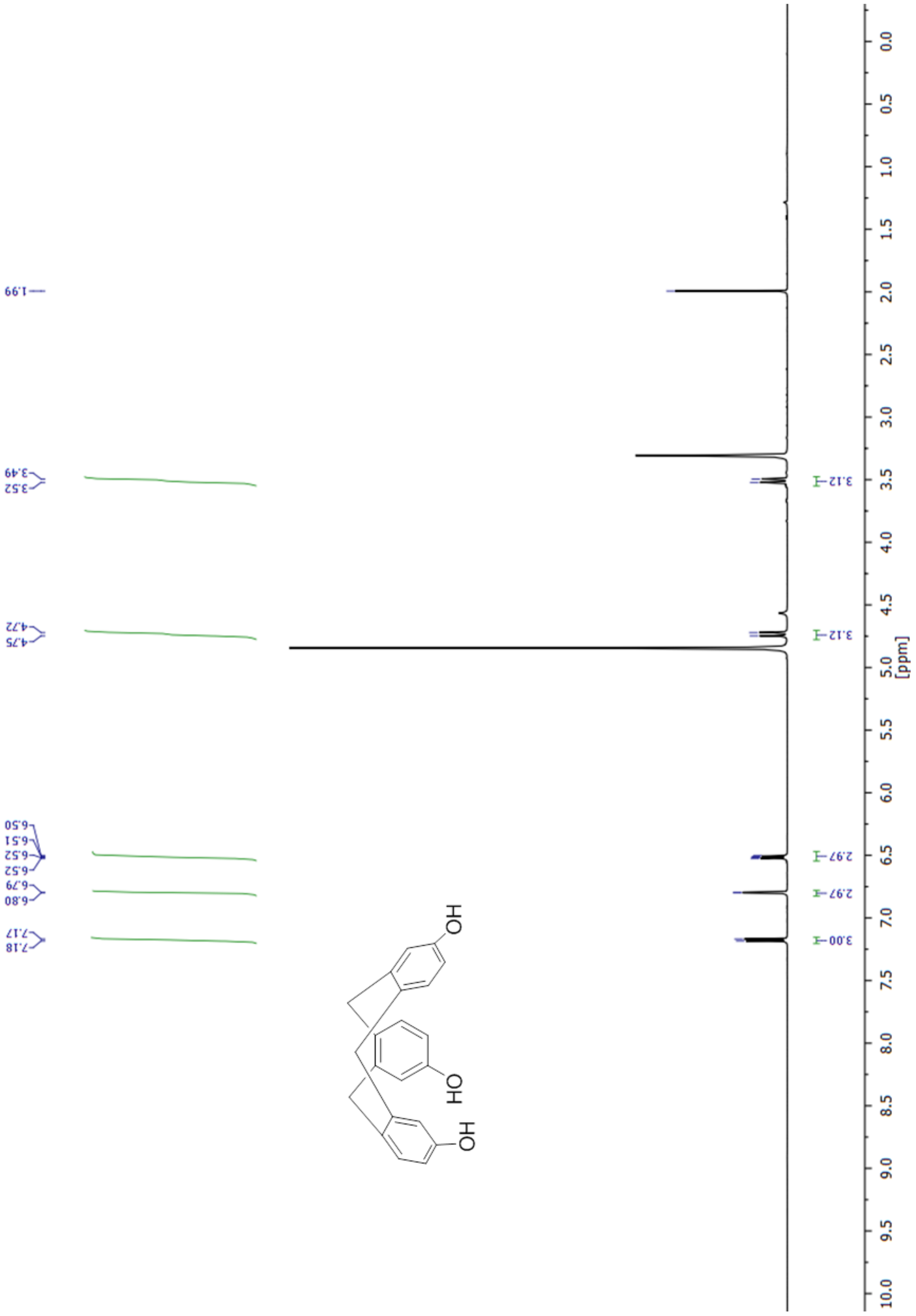

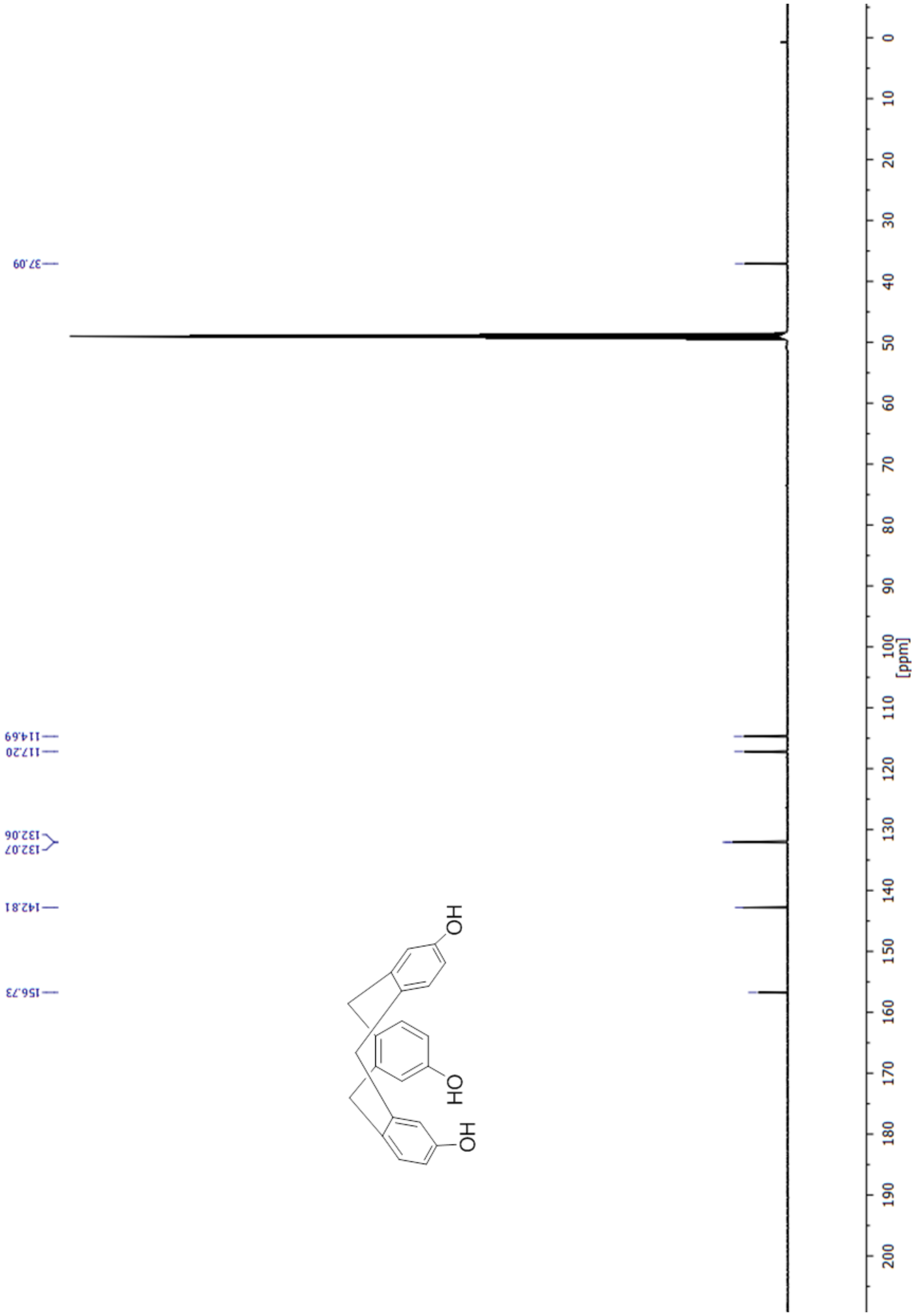

37.09
114.69
117.20
132.06
132.07
142.81
156.73
OH
OH
HO
[ppm]

The signals marked with an asterisk are due to impurities that derive from the HPLC separation.

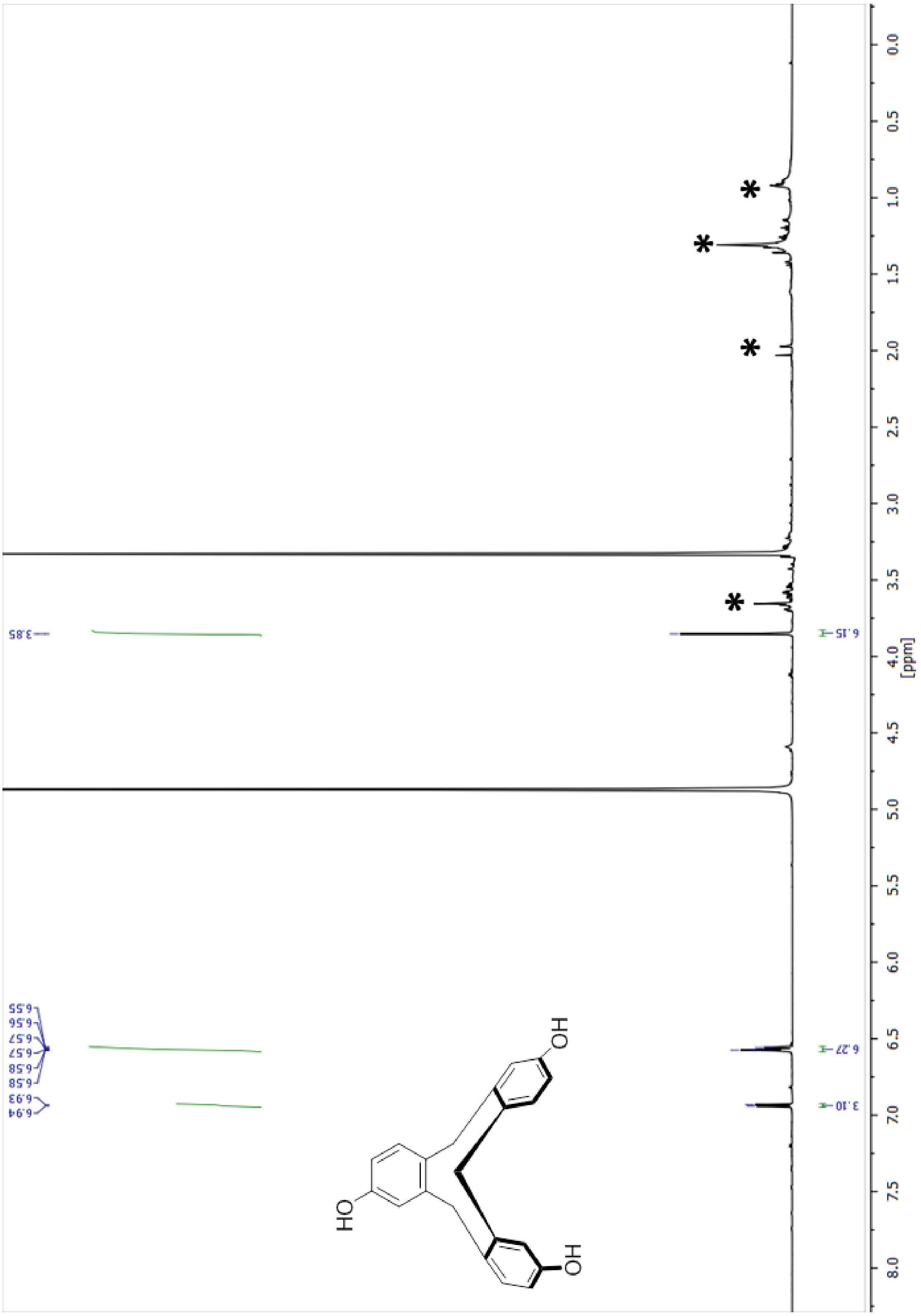

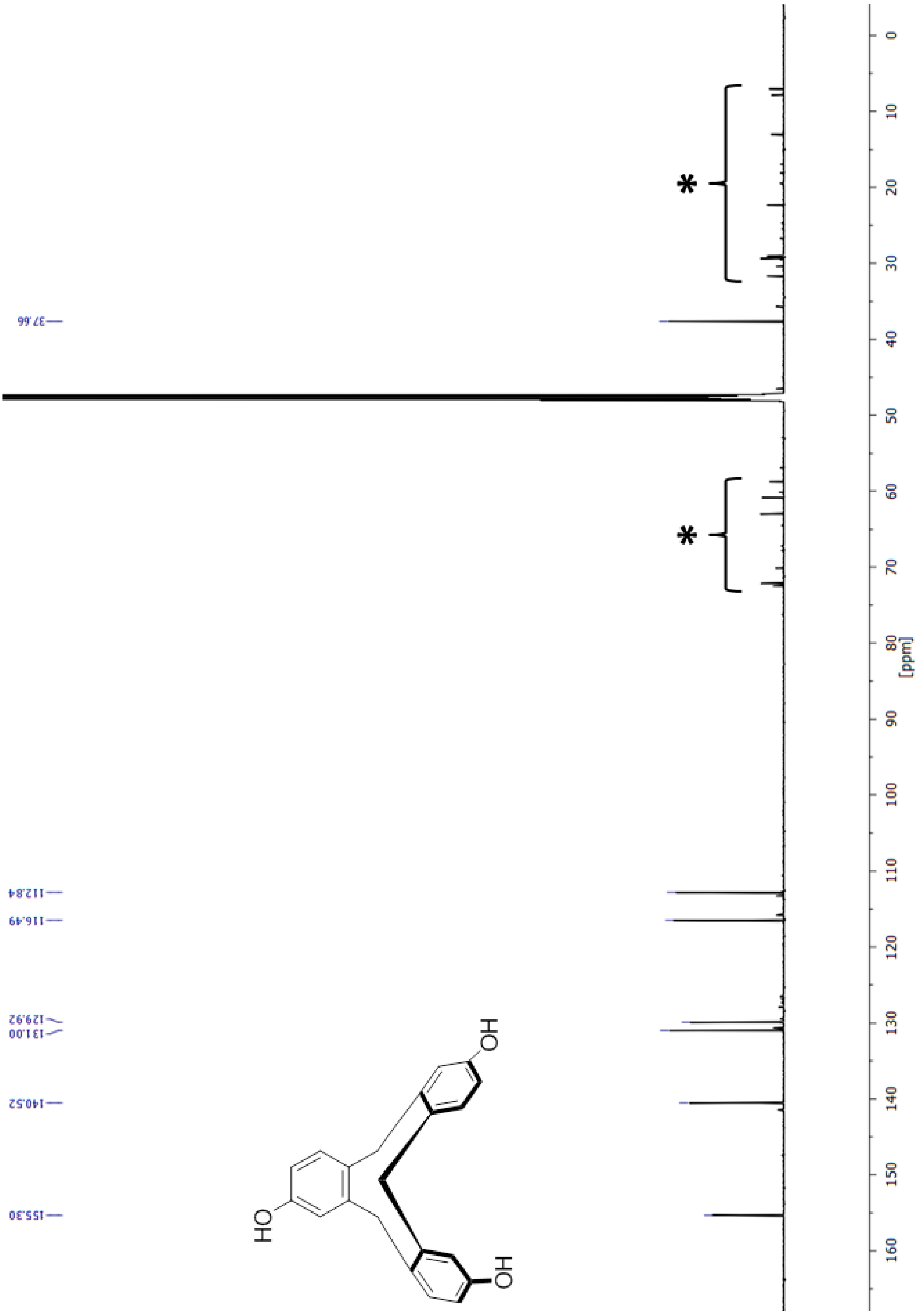
37.66
112.84
116.49
129.92
131.00
140.52
155.30
*
*
[ppm]
HO
OH
OH
